# Low concentration atropine eye drops and progression of myopia in children: multicentre placebo controlled, double masked, randomised trial in the UK (CHAMP-UK)

**DOI:** 10.1136/bmj-2025-086698

**Published:** 2026-06-11

**Authors:** Augusto Azuara-Blanco, Nicola S Logan, Emma McConnell, Stephanie Kearney, Gaynor Kirk, Susie Jones, Cliona McDowell, Lynn Murphy, Gerard O’Hanlon, Margaret McFarland, Sally Painter, Brinda Muthusamy, Shariar Nabili, Jennifer Preston, Ian Flitcroft, James Loughman, David Mackey, Samantha Lee, Annegret Dahlmann-Noor, Nathan Congdon, Ruth E Hogg, Chris J Hammond, Kathryn Saunders, Peter M Allen, Niall Strang, Mike Clarke

**Affiliations:** 1 Centre for Public Health, Queen’s University Belfast, Belfast, UK; 2 School of Optometry, Aston University, Birmingham, UK; 3 Northern Ireland Clinical Research Facility, Belfast, Ireland, UK; 4 Department of Vision Sciences, Glasgow Caledonian University, Glasgow, UK; 5 Vision and Hearing Sciences Research Centre, Anglia Ruskin University, Cambridge, UK; 6 Northern Ireland Clinical Trials Unit, Belfast, Ireland, UK; 7 Department of Pharmacy, Belfast Health and Social Care Trust, Belfast, Ireland, UK; 8 Birmingham Women and Children’s NHS Foundation Trust, Birmingham, UK; 9 Addenbrooke’s Hospital, Cambridge, UK; 10 NHS Lanarkshire, UK; 11 Institute of Translational Medicine, University of Liverpool, Liverpool, UK; 12 Centre for Eye Research Ireland, Technological University Dublin, Dublin, Ireland; 13 Optometry School, Technological University Dublin, Dublin, Ireland; 14 Lions Eye Institute, University of Western Australia, Perth, Australia; 15 NIHR Moorfields Biomedical Research Centre, London, UK; 16 Orbis International, New York, USA; 17 Zhongshan Ophthalmic Centre, Sun Yat-sen University, Guangzhou, China; 18 King’s College London, London, UK; 19 School of Biomedical Sciences, Ulster University, Coleraine, UK

## Abstract

**Objectives:**

To evaluate the efficacy and safety of low concentration atropine eye drops for reducing progression of myopia in children in the UK.

**Design:**

Multicentre, double masked, superiority, placebo controlled, randomised trial.

**Setting:**

National Health Service hospital eye services and academic institutions at five UK centres.

**Participants:**

289 children aged 6-12 years with myopia between −0.50 and −10.0 dioptres (D). Participants were allocated in ratio of 2:1 to atropine or placebo.

**Interventions:**

One eye drop of preserved atropine 0.01% or placebo daily for two years.

**Main outcome measures:**

The primary outcome was spherical equivalent refractive error of both eyes measured by autorefractor under cycloplegia after two years. Secondary outcomes included change in axial length, best corrected distance and near visual acuity, reading speed, pupil diameter, spectacle correction, adverse event rates, quality of life, and tolerability. Outcomes were collected every six months. An electronic monitoring system was used to assess adherence.

**Results:**

192 participants were included in the atropine group and 97 in the placebo group, with an average age of 9.3 years (standard deviation (SD) 1.7 years). 207 (72%) reported white ethnicity, 161 (56%) were girls, and the mean level of myopia was −2.87 D (SD 1.71 D). A total of 235 (81%) participants completed the study, with primary outcome data available for 230 (80%) participants: 151 (79%) in the atropine group and 79 (81%) in the placebo group. Atropine eye drops were more effective than placebo in reducing myopia progression (mean difference 0.33 D, 95% confidence interval (CI) 0.17 to 0.49 D, P<0.001). Prespecified subgroup analyses did not show differences according to age, ethnicity, sex, or severity of myopia. Changes in central axial length were significantly less in the atropine group versus placebo group: mean difference 0.14 mm (95% CI 0.07 to 0.21, P<0.001). There were no differences in other secondary outcomes, except pupil diameter, which was greater in the atropine group (0.36 mm, 95% CI 0.17, 0.55, P<0.001), and no differences in frequency of adverse events or in tolerability measures. No serious adverse events were related to the trial drugs.

**Conclusions:**

Low concentration atropine (0.01%) eye drops significantly reduced progression of myopia and were well tolerated compared with placebo in children in the UK.

**Trial registration:**

ISRCTN registry ISRCTN99883695, ClinicalTrials.gov NCT03690089.

**Figure:**
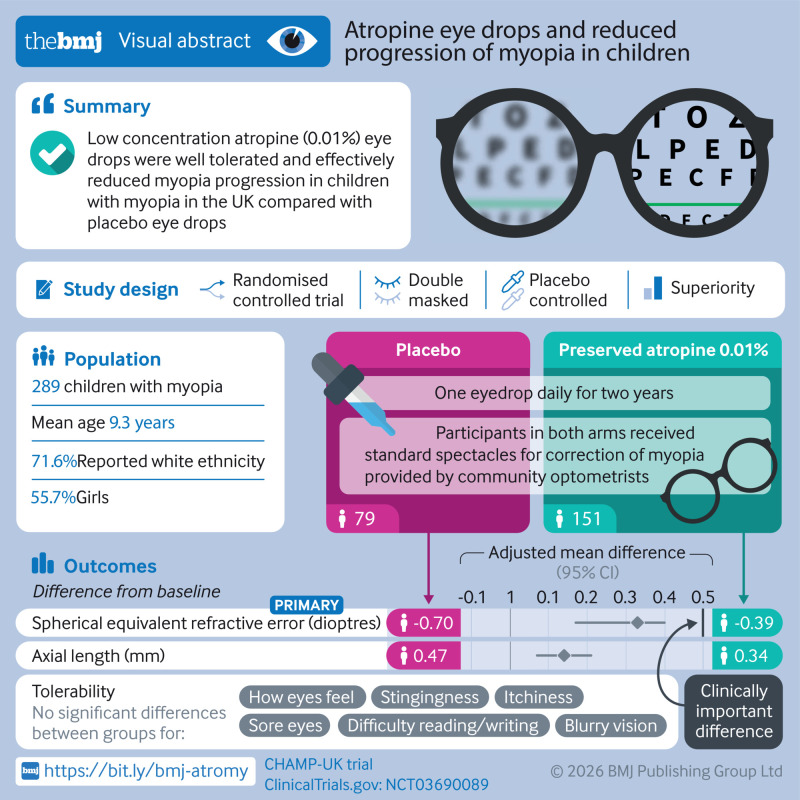


## Introduction

In Europe, the prevalence of myopia has increased noticeably over the past few decades.^1^ ^2^ ^3^ ^4^ ^5^ ^6^ This condition seems to be occurring at a younger age, with an average increase in severity of about 1 dioptre (D) among European derived populations in one generation.^3^ ^7^ In the UK, the prevalence of myopia in children has more than doubled since the 1960s.^6^ In 12-13 year olds in the UK, the prevalence of myopia is about 16%,^6^ and in people born in 1965-70 it is around 30%.^8^ In the US, prevalence has increased from 25% to 42% in a generation^7^ and is reported to be as much as 50% in Sweden.^9^

Most people with myopia have normal visual acuity when appropriately corrected with spectacles or contact lenses, but myopia has important educational, financial, and psychological consequences and increases the risk of visual impairment in adult life.^10^ ^11^ ^12^ Myopia is a risk factor for myopic maculopathy, retinal detachment, cataract, and glaucoma,^11^ ^13^ ^14^ and the risk increases with the degree of myopia. These conditions may lead to visual loss, in some cases irreversibly. Thus, interventions that reduce the progression and severity of myopia in childhood will have long term health and economic benefits. Strategies to control progression of myopia are particularly meaningful in the context of World Health Organization initiatives to eliminate preventable causes of blindness.^15^

Various optical and drug interventions are proven to reduce the progression of myopia in childhood. A recent Cochrane review found that high concentration atropine (≥0.5%) may be the most effective treatment option, but the side effects (photophobia, blurred vision at near reading distance, and allergic reactions) render it unsuitable for routine clinical interventions to control myopia.^16^ The review highlighted uncertainties about the comparative effectiveness of available interventions. Although most trials using atropine for myopia control have been conducted in children of Chinese ethnicity,^17^ ^18^ ^19^ ^20^ ^21^ several recent studies have examined low concentration atropine in western populations, with variable results.^22^ ^23^ ^24^ ^25^ In addition, atropine eye drops are not available in the UK National Health Service (NHS) for treating myopia. In the CHAMP-UK (Childhood Atropine for Myopia Progression in the UK) study we evaluated the efficacy, safety, and mechanism of action of low concentration atropine (0.01%) in UK children with myopia aged 6 to 12 years.

## Methods

CHAMP-UK was a multicentre, randomised, double masked, placebo controlled, superiority trial, with participants allocated in a ratio of 2:1 to atropine or placebo. The trial protocol has been published previously,^26^ and the trial was prospectively registered before enrolment of the first participant.

### Setting and participants

The study was performed at clinical research facilities in five academic departments of medical or optometry schools and NHS trusts in Northern Ireland (Belfast), England (Birmingham, Cambridge, London), and Scotland (Glasgow). Children were eligible to participate in the study if they were aged 6-12 years and had myopia of −0.50 D or greater (spherical equivalent refractive error) and a best corrected distance visual acuity of 0.20 logMAR (logarithm of the minimum angle of resolution) or better in both eyes. We excluded children with other major ocular or systemic morbidities (ie, any disease that might compromise vision or require ocular surgery, such as diabetes mellitus), myopia of −10 D or greater in either eye, astigmatism of 2 D or greater in either eye, amblyopia, important health problems that could compromise the ability to attend research visits or complete the trial, other factors that might compromise the ability to attend the research appointments, poor English literacy, including the parents, enrolment in other interventional trials, allergy or hypersensitivity to atropine or excipients, and previous or current use of atropine or orthokeratology contact lenses, other contact lenses, or spectacles to control myopia.

### Recruitment, randomisation, and allocation

Recruitment was through community optometrists and paediatric ophthalmologists who informed parents of children with myopia about the trial. After parental consent had been obtained, children were screened and, if eligible, enrolled and randomised. Randomisation (2:1) was computer generated using a minimisation algorithm to ensure balanced allocation of participants across the two treatment groups. A participant’s allocation was fully concealed from everyone involved in recruiting participants to the trial. Minimisation was by centre, ethnic background (white or non-white), and severity of myopia (less than −3.00 D in either eye or −3.00 D or more in the eye with the most severe myopia). The unit of randomisation was the participant (not the eye). Sealed envelope (sealedenvelope.com) generated the randomisation list, with group allocation visible only to the senior statistician at the Northern Ireland Clinical Trials Unit. Local researchers accessed the automated randomisation system to obtain the kit number for each participant. Investigators (chief, principal, and co-investigators), study monitors, and study participants were all masked throughout the study.

### Study interventions

The intervention group was assigned to receive preserved atropine sulfate 0.01% eye drops once daily at bedtime in both eyes for two years. The control group received placebo eye drops on the same dosing schedule, with the same preservative (benzalkonium chloride 0.01% weight/volume in sterile water) and pH. Bottles containing atropine and placebo were identical to ensure masking of participants and investigators to study group assignment. Participants attended the research centre every six months (two weeks either way) across the two year follow-up.

### Outcomes

The primary outcome was spherical equivalent refractive error (calculated as the spherical power plus half the cylindrical power, an index of myopia severity) of both eyes 24 months after enrolment, measured by autorefractor under cycloplegia, and adjusted for baseline. Each site used the same type of autorefractor throughout the study (Grand Seiko Binocular Auto-refractor/Keratometer WR-5100 K, WAM-5500 or Shin-Nippon Accuref K-900; Grand Seiko, Hiroshima, Japan). Participating children received one or two drops of 1% cyclopentolate hydrochloride in each eye at least 20 minutes before autorefraction, with another drop instilled if full cycloplegia had not been achieved. Cycloplegia was checked by absence of light reflex and a push-up amplitude of accommodation less than 2 D. The autorefractor calculates the spherical equivalent refractive error as the mean of five valid readings.

Secondary outcomes included central axial length (measured with partial coherence interferometry, and at each site the same instrument was used throughout the study) and the IOLMaster (Carl Zeiss Meditec, Dublin, CA) or Haag-Streit Lenstar LS900 (Haag-Streit Group, Switzerland) best corrected distance visual acuity (monocular and binocular), monocular and binocular near visual acuity (early treatment diabetic retinopathy study chart^27^), reading speed (Wilkins rate of reading test),^28^ pupil diameter (by autorefractometer under photopic conditions), spectacle correction power, adverse event rates, quality of life (Euro-Qol five dimensions, youth, five levels (EQ-5D-Y-5L)),^29^ and tolerability (assessed by questionnaire) at two years. Tolerability was determined using a 4 point scale to quantify, from the participant’s point of view, ocular irritation or stinging, photophobia, and difficulties reading and writing. The study also explored possible mechanisms of action of atropine,^30^ ^31^ ^32^ ^33^ ^34^ ^35^ which will be published separately. The parents or guardians of participants will be contacted at the five year time point (three years after cessation of eye drops) to evaluate their child’s refractive error and possible adverse events.

Adherence was assessed using electronic monitoring with a medical events monitoring system (MEMS) device (AARDEX Group, Switzerland). The MEMS Cap is a plastic container with a screw top in which the eye drop bottle can be stored until needed.^36^ When the top is unscrewed, the device electronically records the date and time. These data are downloaded, analysed, and taken as a surrogate for the drug being administered. We considered use of at least 80% of prescribed doses as meeting the requirements for adherence.

### Sample size

We anticipated that progression in the control group and the effect of atropine eye drops in a UK population would be smaller than reported in Chinese populations^19^ and assumed that atropine would reduce the two year progression of myopia by at least 40% from a control mean of −0.8 D (equivalent to a mean difference of −0.32 D). With a standard deviation (SD) of 0.7, an intraclass correlation coefficient between the two eyes of 0.9, and a variation inflation factor of 1.9, we determined that we would need 228 participants (456 eyes). Considering a dropout rate of 15% and that 10% of recruited children could be Chinese, we increased the number required to a total of 289 participants: 193 in the atropine group and 96 in the placebo group, to detect this difference in the non-Chinese UK population with 90% power. The justification is that in a study of 400 Chinese children in which the effect of 24 months usage of 1% atropine eye drops was evaluated, myopia progression was −1.20 D (SD 0.69 D) in the placebo control group and −0.28 (0.92) D in the atropine group. We have assumed that progression of myopia and efficacy of atropine will be less in UK children than in Chinese children and less with 0.01% atropine eye drops. Progression of myopia in untreated children was estimated from the control groups of randomised controlled trials of myopia control interventions. The following progression data have been reported in children: in Singapore, −1.28 (SD 0.78) D after two years (control was single vision spectacles^37^); in Hong Kong, −1.26 (SD 0.74) D after two years (control was single vision spectacles^38^); in the ATOM study in Singapore (Chinese race),^39^ −1.20 (SD 0.69) D after two years (control was placebo drops); in the US, the COMET trial (children of mixed races, with white being the most common ethnicity),^40^ 1.32 (SD 0.04) D after three years (control was single vision spectacles); and in an observational study from the UK, 1.14 D after three years.^2^

### Adverse events and safety reporting

Serious adverse events related to the use of the study drugs in the trial were reported in accordance with the guidance from the European Clinical Trials Directive 2001/20/EC (https://ec.europa.eu/health/human-use/clinical-trials/directive_en).

### Quality control and data management

The Clinical Trials Unit that oversaw the study conducted quality control. The unit also ensured consistent data entry, verification, and management across the centres. A manual was used for the clinical procedures to ensure standardisation and consistency of data collection between and within clinical sites. Study data were entered into a web based Clinical Trial Database (MACRO release 4.9.1, Clinical Data Management System, Ennov SAS, Paris) and processed electronically.

### Statistical analysis

For the primary analysis, participants were analysed according to their randomisation group (intention-to-treat principle). Intention to treat includes all randomised participants analysed in their assigned group regardless of adherence or dropout. Per protocol analysis is defined as participants with overall adherence to the study drug of at least 80% using MEMs data. We considered P<0.05 to be statistically significant. Baseline characteristics were summarised as mean and SD, median and interquartile range (IQR), or numbers and proportions (%), as appropriate, depending on the scale of measurement and distribution. Analyses were performed with Stata Statistical Software, version 15.1 (StataCorp, College Station, TX).

The principal analysis was based on complete case data without imputation for those who dropped out of the study before 24 months. The results of the primary outcome (spherical equivalent refractive error at month 24) from both eyes were used and adjusted for baseline spherical equivalent refractive error. We compared the atropine and control groups using generalised estimating equations and 95% confidence intervals (CIs) to allow for the correlation between eyes within a participant. Secondary analysis of the primary outcome was adjusted for baseline spherical equivalent refractive error, minimisation variables (ie, site and ethnicity), baseline age, and history of myopia in at least one parent. The impact of missing data for the primary outcome was assessed by sensitivity analyses by imputing extreme values of change within treatment arm (ie, from smallest to the largest change from the baseline time point to 24 months without use of other myopia management products).

For other secondary outcomes such as uniocular best corrected distance visual acuity, uniocular near visual acuity, pupil diameter, and central axial length, we pooled outcomes from both eyes in combined analysis using generalised estimating equations to allow for the correlation between eyes within a participant. We used the independent *t* test to determine the significance of difference in tolerability between the atropine and control groups. Analysis of covariance was used for outcomes such as reading speed, binocular best corrected distance visual acuity, binocular near visual acuity, and EQ-5D, to adjust for the corresponding baseline measures. χ^2^ tests were used to test the difference in the proportions between the groups for the categorical outcomes of spectacle correction and frequency of spectacle correction. Repeated measures mixed models were used when analysing outcomes over time. Subgroup analyses were performed on the primary outcome using 99% confidence intervals and interaction terms (treatment group by subgroup) for the several prespecified subgroups: age (6-9 and 10-12 years at randomisation), ethnic background (white versus non-white), severity of myopia (less than −3 D in either eye versus −3 D or greater myopia), iris colour (dark brown and other, including light brown, green, blue, and grey), and sex (boy or girl). The rationale for the subgroup analyses was to analyse the factors that are known to influence rate of myopia progression.^1^ ^11^ ^17^ A detailed statistical analysis plan was completed before we began data analysis. Post hoc additional analyses were undertaken to ascertain the proportion of participants with myopia progression less than 0.25 D and with myopia progression of greater than 0.25 D, 0.50 D, 0.75 D, and 1 D, reporting P values from χ^2^ test.

### Monitoring and protocol adherence

On-site monitoring complied with the principles of Good Clinical Practice. On-site monitoring visits during the trial were conducted every six months for each site, which included checking the accuracy of entries on participants’ case report forms against the source documents, adherence to the protocol, study procedures, and good clinical practice.

We defined a protocol deviation as an incident that deviated from the normal expectation of a particular part of the trial process.

### Patient and public involvement

CHAMP-UK addresses one of the research priorities identified by the James Lind Alliance: How can the progression of myopia be prevented?^41^ In developing the design of this trial, we actively sought the input of parents of children with myopia. Some of the parents have myopia themselves. Parents welcomed a trial to identify a treatment that could reduce myopia progression and stressed that safety was a major consideration. In addition, we conducted an electronic survey with 35 parents of children with myopia involved in other research studies, among whom 28 (80%) responded. All were supportive of this trial; 93% wanted to have confirmation of the safety of the intervention.

During the design and conduct of the trial and preparation of participant-facing study materials, we had input from Jennifer Preston, patient and public involvement manager for the National Institute for Health and Care Research Alder Hey Clinical Research Facility, and an advisory group of two parents with experience of looking after a child with myopia, who attended project steering group meetings.

## Results

**Fig 1:**
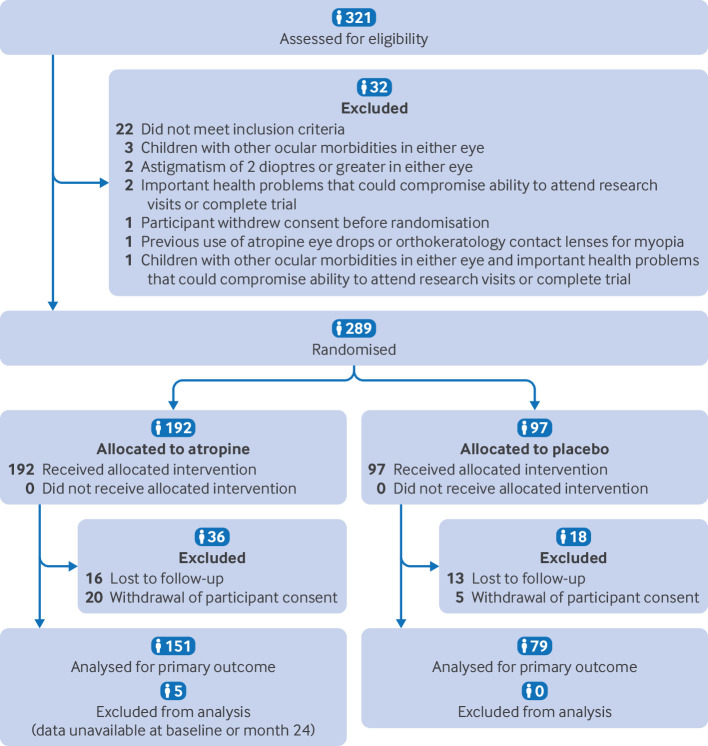
Study flow diagram of study participant enrolment, randomisation, and follow-up

Participants were recruited between June 2019 and February 2022. This recruitment period was extended owing to the covid-19 pandemic, with the last 24 month follow-up visit occurring in February 2024. We enrolled 289 participants: 192 (66%) in the atropine group and 97 (34%) in the placebo group. The average age of participants was 9.3 (SD 1.7 years) and 207 (72%) reported white ethnicity (table 1). A total of 235 participants (81%) completed the two year follow-up, with primary outcome data available for 230 participants (80%): 151 (79%) in the atropine group and 79 (81%) in the placebo group (table 2). Randomisation remained at about 2:1 (fig 1).

**Table 1:** Baseline characteristics of children with myopia by treatment group. Values are number (percentage) unless stated otherwise

Characteristics	Treatment group		Total (n=289)
	0.01% atropine sulfate (n=192)	Placebo (n=97)	
Minimisation factors:			
White	138 (72)	71 (73)	209 (72)
Non-white	54 (28)	26 (27)	80 (28)
Severity of myopia (D):			
−3.00 or more	81 (42)	42 (43)	123 (43)
Less than −3.00	111 (58)	55 (57)	166 (57)
Sex:			
Boy	90 (47)	38 (39)	128 (44)
Girl	102 (53)	59 (61)	161 (56)
Mean (SD) age (years)	9.3 (1.7)	9.3 (1.7)	9.3 (1.7)
Mean (SD) weight (kg)	36.4 (11.2)	35.7 (9.7)	36.1 (10.7)
Mean (SD) height (cm)	141.2 (12.8)	141.5 (11.4)	141.3 (12.3)
Ethnicity:			
White	136 (701)	71 (73)	207 (72)
Mixed/multiple ethnic groups	13 (7)	6 (6)	19 (7)
Asian/Asian British (excluding Chinese)	26 (13)	9 (9)	35 (12)
Black/African/Caribbean/black British	2 (1)	1 (1)	3 (1)
Chinese	8 (4)	3 (3)	11 (4)
Other	7 (4)	7 (7)	14 (5)
Parental myopia*:			
Yes†	162 (84)	83 (86)	245 (85)
No	25 (13)	12 (12)	37 (13)
Unknown	5 (2)	2 (2)	7 (2)
Mean (SD) SER (D):			
Both eyes	−2.96 (1.79) n=189	−2.69 (1.56)	−2.87 (1.71) n=286
Left eye	−2.96 (1.83) n=190	−2.72 (1.59)	−2.88 (1.75) n=287
Right eye	−2.99 (1.82) n=190	−2.66 (1.59)	−2.88 (1.75) n=287
Mean (SD) axial length (mm):			
Both eyes	24.58 (1.05)	24.39 (0.92)	24.51 (1.01)
Left eye	24.57 (1.06)	24.40 (0.96)	24.51 (1.03)
Right eye	24.59 (1.05)	24.39 (0.92)	24.52 (1.01)
Mean (SD) EQ-5D-Y-visual analogue scale‡	95.6 (7.3)	95.0 (8.1)	95.4 (7.6)
Mean (SD) monocular BCdVA (logMAR):			
Both eyes	84.0 (3.7)	83.8 (3.6)	84.0 (3.7)
Left eye	83.9 (4.0)	83.9 (3.9)	83.9 (3.9)
Right eye	84.2 (3.9)	83.7 (3.7)	84.0 (3.8)
Mean (SD) binocular BCdVA (logMAR)	86.1 (4.0)	86.3 (3.9)	86.2 (4.0)
Mean (SD) monocular near visual acuity:			
Both eyes	68.0 (4.0)	67.8 (4.2) n=96	67.9 (4.1) n=288
Left eye	68.2 (4.3)	68.1 (4.3) n=96	68.1 (4.3) n=288
Right eye	67.8 (4.2)	67.4 (4.5) n=96	67.7 (4.3) n=288
Mean (SD) binocular near visual acuity	70.4 (3.7)	70.5 (4.3) n=96	70.4 (3.9) n=288
Mean (SD) reading speed (words/min)	106.5 (29.7)	104.8 (27.8)	105.9 (29.0)
Mean (SD) pupil diameter (mm):			
Both eyes	6.0 (0.92)	6.0 (0.92)	6.0 (0.92)
Left eye	6.0 (0.96)	6.0 (0.97)	6.0 (0.96)
Right eye	6.0 (0.92)	6.0 (0.93)	6.0 (0.93)
Spectacle correction:			
Yes	192 (100)	95 (98)	287 (99)
No	0 (0)	2 (2)	2 (1)
Frequency of spectacle correction:			
All day	149 (78)	77 (81)	226 (79)
Sometimes	43 (22)	18 (19)	61 (21)
Contact lens use:			
Yes	13 (7)	4 (4)	17 (6)
No	179 (93)	93 (96)	272 (94)
Frequency of contact lens use:			
All day	4 (31)	0 (0)	4/17 (24)
Sometimes	9 (69)	4 (100)	13/17 (76)

D=dioptre; BCdVA=best corrected distance visual acuity; EQ-5D-Y=EuroQol-5 dimensions-youth; SD=standard deviation; SER=spherical equivalent refractive error.

* Based on parents’ current spectacle prescription.

† One or both parents with myopia.

‡ Not weighted.

**Table 2:** Primary outcome and secondary outcomes in children aged 6-12 years with myopia by treatment group. Values are mean (SD) unless stated otherwise

Main outcomes	Treatment group			Adjusted*			Adjusted†	
	0.01% atropine sulfate; No in group	Placebo; No in group		Mean difference (95% CI)	P value		Mean difference (95% CI)	P value
**Primary outcome: change in SER from baseline to 24 months**								
Intention to treat‡	−0.39 (0.63); n=151	−0.70 (0.67); n=79		0.31 (0.14 to 0.48)	<0.001		0.33 (0.17 to 0.49)	<0.001
Per protocol‡	−0.41 (0.59); n=102	−0.75 (0.56); n=57		0.33 (0.15 to 0.52)	<0.001		0.38 (0.20 to 0.56)	<0.001
**Secondary outcomes**								
Reading speed§:								
Intention to treat	133.4 (30.2); n=156	133.6 (29.8); n=79		−1.70 (−7.10 to 3.69)	0.53			
Per protocol	132.2 (29.4); n=106	134.4 (26.6); n=57		−1.95 (−7.76 to 3.86)	0.51			
Uniocular BCdVA‡:								
Intention to treat	55.3 (4.43); n=156	55.3 (3.28); n=79		−0.08 (−1.02 to 0.87)	0.87			
Per protocol	56.0 (3.71); n=106	55.7 (2.97); n=57		0.41 (−0.46 to 1.28)	0.36			
Binocular BCdVA§:								
Intention to treat	58.1 (3.55); n=156	57.7 (3.40); n=79		0.60 (−0.11 to 1.30)	0.097			
Per protocol	58.3 (3.42); n=106	58.2 (3.17); n=57		0.71 (−0.07 to 1.49)	0.073			
Central axial length‡:								
Intention to treat	24.9 (1.04); n=155	24.9 (0.88); n=79		−0.12 (−0.20 to −0.04)	0.003		−0.14 (−0.21 to −0.07)	<0.001
Per protocol	24.9 (1.01); n=106	24.9 (0.90); n=57		−0.11 (−0.21 to −0.01)	0.034		−0.15 (−0.24 to −0.06)	0.001
Uniocular near visual acuity‡:								
Intention to treat	69.1 (4.57); n=157	69.6 (4.08); n=79		−0.66 (−1.75 to 0.44)	0.24			
Per protocol	69.5 (3.67); n=106	69.8 (3.97); n=57		−0.22 (−1.24 to 0.80)	0.67			
Binocular near visual acuity§:								
Intention to treat	71.9 (3.82); n=157	71.7 (3.91); n=79		0.17 (−0.73 to 1.07)	0.71			
Per protocol	72.0 (3.71); n=106	72.1 (3.96); n=57		0.24 (−0.81 to 1.30)	0.65			
Pupil diameter‡:								
Intention to treat	6.43 (0.96); n=156	6.02 (0.87); n=79		0.36 (0.17 to 0.55)	<0.001			
Per protocol	6.62 (0.82); n=106	6.08 (0.93); n=57		0.59 (0.38 to 0.80)	<0.001			
Amplitude of accommodation‡:								
Intention to treat	13.4 (3.5); n=153	14.8 (3.4); n=78		−1.0 (−2.0 to −0.1)	0.03			
Per protocol	12.9 (3.5); n=103	14.6 (3.4); n=56		−1.4 (−2.5 to −0.3)	0.01			
Spectacle correction (No (%))¶:								
Intention to treat	154 (99)	76 (96)						
Per protocol	106 (100)	55 (96.5)						
Frequency of spectacle correction (all day) (No (%))¶:								
Intention to treat	127 (82.5)	62 (82)						
Per protocol	92 (87)	45 (82)						
Frequency of spectacle correction (sometimes) (No (%))¶:								
Intention to treat	27 (17.5)	14 (18)						
Per protocol	14 (13)	10 (18)						
EQ-5D-Y visual analogue scale§:								
Intention to treat	92.5 (10.3); n=156	93.9 (8.25); n=79		−1.62 (−4.01 to 0.76)	0.18			
Per protocol	93.6 (8.46); n=106	94.0 (7.85); n=57		−0.22 (−2.69 to 2.24)	0.86			
Tolerability**:								
How eyes feel today:								
Intention to treat	3.72 (0.50); n=156	3.77 (0.45); n=78		−0.04 (−0.18 to 0.09)	0.51			
Per protocol	3.72 (0.51); n=105	3.81 (0.44); n=57		−0.08 (−0.24 to 0.07)	0.30			
Stingy:								
Intention to treat	3.48 (0.76); n=149	3.43 (0.85); n=74		0.04 (−0.18 to 0.27)	0.69			
Per protocol	3.47 (0.76); n=105	3.45 (0.83); n=56		0.02 (−0.24 to 0.28)	0.88			
Itchiness:								
Intention to treat	3.47 (0.81); n=157	3.53 (0.83); n=78		−0.05 (−0.28 to 0.17)	0.63			
Per protocol	3.56 (0.77); n=106	3.54 (0.83); n=57		0.01 (−0.24 to 0.27)	0.92			
Blurry vision:								
Intention to treat	3.74 (0.66); n=156	3.69 (0.83); n=77		0.06 (−0.14 to 0.25)	0.58			
Per protocol	3.75 (0.68); n=106	3.64 (0.88); n=56		0.10 (−0.14 to 0.35)	0.41			
Sore eyes:								
Intention to treat	3.29 (0.88); n=157	3.45 (0.88); n=78		−0.16 (−0.40 to 0.08)	0.19			
Per protocol	3.25 (0.90); n=106	3.46 (0.87); n=57		−0.20 (−0.49 to 0.09)	0.17			
Difficulty reading/writing:								
Intention to treat	3.69 (0.76); n=157	3.69 (0.83); n=78		0.002 (−0.21 to 0.21)	0.99			
Per protocol	3.72 (0.73); n=106	3.67 (0.85); n=57		0.05 (−0.20 to 0.30)	0.69			

Amplitude of accommodation was an exploratory outcome.

BCdVA=best corrected distance visual acuity; CI=confidence interval; SD=standard deviation; SER=spherical equivalent refractive error.

* Adjusted for baseline.

† Adjusted for baseline SER, minimisation variables (ie, site and ethnicity), age, and history of myopia in at least one parent.

‡ Values were calculated with data from both eyes. Mean difference estimate and P value from generalised estimating equations.

§ Mean difference estimate and P value from analysis of covariance.

¶ Risk difference and P value from the χ^2^ test.

** Mean (SD) and P value from independent *t* test. No adjustment as tolerability was not measured at baseline.

For the primary outcome, atropine 0.01% eye drops reduced myopia progression compared with placebo, both in the intention-to-treat analysis (mean difference 0.33 D, 95% CI 0.17 to 0.49, P<0.001) and in the per protocol analysis (80% adherence according to MEMS device) (mean difference 0.38 D, 0.20 to 0.56, P<0.001), adjusting for baseline myopia, site, ethnicity, age, and history of myopia in at least one parent (table 2). In the post hoc sensitivity analysis, more participants had stable myopia (a change in level of myopia less than 0.25 D) in the atropine group (n=61, 40%) than in the control group (n=15, 19%, P=0.003), and there were fewer participants with significant myopia progression (change more than 0.50 D) in the atropine group (n=64, 42%) than in the control group (n=50, 63%, P=0.007). The number of children with progression of 1 D was substantially reduced in the atropine versus control group (16 (16%) *v* 24 (42%)) (table 3). Sensitivity analyses for missing data showed a smaller non-significant between group mean difference (0.16, −0.06 to 0.37, P=0.16) when imputing the largest (worst) changes of −2.31 for atropine and −2.04 for placebo. In contrast, imputing the smallest (best) changes of 1.4 for atropine and 0.52 for placebo resulted in a larger significant between group mean difference (0.47, 0.26 to 0.68, P<0.001).

**Table 3:** Proportion of children (6-12 years) by category of myopia progression over two years. Values are number (percentage) unless stated otherwise

Variables	Treatment group	
	0.01% atropine sulfate	Placebo
**Intention to treat**	n=151	n=79
<0.25 D	61 (40)	15 (19)
0.25 D to 0.5 D	26 (17)	14 (18)
0.5 D to 1 D	39 (26)	20 (25)
>1 D	25 (17)	30 (38)
**Per protocol**	n=102	n=57
<0.25 D	42 (41)	10 (17.5)
0.25 D to 0.5 D	15 (15)	9 (16)
0.5 D to 1 D	29 (28)	14 (25)
>1 D	16 (16)	24 (42)

P=0.001 based on χ^2^ test for intention-to-treat analysis and P=0.001 for per protocol analysis.

D=dioptre.

Central axial length growth in the atropine group was less than in the placebo group, both in the intention-to-treat analysis adjusting for baseline, site, ethnicity, age, and history of myopia in at least one parent (mean difference 0.14 mm, 95% CI 0.07 to 0.21, P<0.001) and per protocol analysis (mean difference 0.15 mm, 0.06 to 0.24, P=0.001) (table 2).

Except for pupil size (measured in approximately 450 lux light levels), which was greater in the atropine group, and binocular amplitude of accommodation, which was lower in the atropine group, there were no statistically significant differences in other secondary outcomes (table 2). There were no statistically significant between group differences in frequency of adverse events and tolerability measures (table 4 and supplementary table 1). No serious adverse events were deemed related to the treatment.

**Table 4:** Treatment after trial entry of children aged 6-12 years with myopia by treatment group. Values are number (percentage) unless stated otherwise

Variables	Treatment group	
	0.01% atropine sulfate (n=192); No in group	Placebo (n=97); No in group
MEMS data*		
Mean (SD) study drug adherence (%)	80.3 (21.5); n=165	80.3 (21.4); n=89
Adherence:		
Yes	113 (68.5)	62 (70)
No	52 (31.5)	27 (30)
Mean (SD) drug holidays (days)	26.3 (36.5); n=161	25.1 (33.7); n=84
Mean (SD) No of overdosage days	5.0 (9.2); n=164	5.1 (10.2); n=87
**Study drug related reasons for stopping treatment**		
Adverse event	5 (3)	1 (1)
Serious adverse event	1 (0.5)	0 (0)
Expiry date	0 (0)	0 (0)
Participant request	10 (5)	10 (10)
Clinician’s request	0 (0)	0 (0)
Other	0 (0)	0 (0)
**Protocol deviations/violations**		
Eligibility	1 (0.5)	0 (0)
Study drug administration:		
Participant reported†	28 (15)	13 (13)
MEMS data†	92 (48)	43 (44)
Safety reporting (adverse event/serious adverse event)	2 (1)	0 (0)
Visit outside schedule or missed visit	90 (47)	54 (56)
Assessment not done‡	40 (21)	22 (23)
Other	26 (13)	14 (14)
Potential use of expired product	7 (4)	7(7)
**Premature withdrawals**		
Adverse event	0 (0)	0 (0)
Serious adverse event	0 (0)	0 (0)
Protocol deviation	0 (0)	0 (0)
Lost to follow-up	16 (8)	13 (13)
Withdrawal of patient consent	20 (10)	5 (5)
Death	0 (0)	0 (0)
Other	0 (0)	0 (0)
Did not receive allocated treatment§	0 (0)	0 (0)
Received treatment of other group	0 (0)	0 (0)
**Use of other myopia management products¶**	10 (5)	6 (6)
Non-standard/non-single vision contact lenses	2	3
Non-standard/non-single vision lenses	4	2
Atropine obtained from other sources	0	1
Other**	5	2

MEMS=medical events monitoring system.

* Data averaged over total study period.

† If drops were missed ≥20% of the time.

‡ Three assessments in the atropine group were related to collection of the primary outcome data at 24 months.

§ Includes those who received no treatment.

¶ Three participants used two myopia management products. Two participants in the atropine group and four in the placebo group also stopped study drug permanently. One participant in the atropine group withdrew consent.

** Includes five participants using myopia control spectacle lenses and two using orthokeratology contact lenses.

Subgroup analyses of the primary outcome did not show any statistically significant differences according to age, ethnicity, sex, iris colour, or severity of myopia at baseline (table 5). Statistically significant differences in spherical equivalent refractive error and axial length were found at other time points in addition to 24 months (see supplementary tables 2 and 3). 

**Table 5:** Primary outcome subgroup analyses of children aged 6-12 years with myopia by treatment group. Values are mean (SD) unless stated otherwise

Outcome by subgroup	Treatment group			Adjusted*	
	0.01% atropine sulfate; No in group	Placebo; No in group		Mean difference (99% CI)	P for interaction†
**Intention-to-treat analysis**					
Age at start of trial (years):					
6-9	−0.59 (0.68); n=82	−0.78 (0.84); n=40		0.17 (−0.13 to 0.48)	0.07
10-12	−0.14 (0.58); n=70	−0.62 (0.51); n=39		0.48 (0.16 to 0.79)	
Ethnicity:					
White	−0.42 (0.59); n=111	−0.72 (0.74); n=58		0.30 (0.03 to 0.56)	0.80
Non-white	−0.29 (0.85); n=41	−0.65 (0.57); n=21		0.35 (−0.09 to 0.79)	
Severity of myopia:					
−3.00 D or more	−0.41 (0.68); n=62	−0.73 (0.62); n=36		0.24 (−0.10 to 0.59)	0.70
Less than −3.00 D	−0.36 (0.67); n=90	−0.68 (0.76); n=43		0.31 (0.01 to 0.61)	
Iris colour:					
Dark brown	−0.27 (0.81); n=49	−0.66 (0.56); n=22		0.38 (−0.04 to 0.80)	0.59
Other‡	−0.44 (0.60); n=103	−0.72 (0.75); n=57		0.28 (0.01 to 0.55)	
Sex:					
Boy	−0.26 (0.66); n=74	−0.69 (0.60); n=30		0.41 (0.05 to 0.76)	0.29
Girl	−0.50 (0.66); n=78	−0.71 (0.75); n=49		0.22 (−0.08 to 0.51)	
**Per protocol analysis**					
Age (years old at start of trial):					
6-9	−0.57 (0.70); n=58	−0.89 (0.62); n=27		0.31 (−0.02 to 0.64)	0.54
10-12	−0.19 (0.48); n=45	−0.62 (0.54); n=30		0.42 (0.09 to 0.75)	
Ethnicity:					
White	−0.45 (0.60); n=79	−0.76 (0.60); n=43		0.31 (0.03 to 0.58)	0.57
Non-white	−0.27 (0.76); n=23	−0.72 (0.58); n=14		0.43 (−0.07 to 0.93)	
Severity of myopia:					
3.00 D or more	−0.43 (0.65); n=41	−0.74 (0.60); n=24		0.27 (−0.11 to 0.65)	0.65
Less than −3.00 D	−0.40 (0.64); n=62	−0.75 (0.60); n=33		0.35 (0.04 to 0.67)	
Iris colour:					
Dark brown	−0.26 (0.68); n=32	−0.72 (0.55); n=16		0.44 (−0.01 to 0.89)	0.46
Other‡ ^c^	−0.47 (0.61); n=71	−0.76 (0.61); n=41		0.28 (−0.003 to 0.57)	
Sex:					
Boy	−0.35 (0.630; n=48	−0.70 (0.63); n=23		0.32 (−0.05 to 0.70)	0.97
Girl	−0.46 (0.65); n=55	−0.78 (0.57); n=34		0.33 (0.01 to 0.65)	
**Central axial length intention-to-treat post hoc analysis**					
Age at start of trial (years):					
6-9	24.9 (1.0); n=83	24.9 (0.9); n=40		−0.12 (−0.26 to 0.01)	0.78
10-12	24.9 (1.1); n=72	25.0 (0.9); n=39		−0.14 (−0.28 to −0.002)	
**Central axial length per protocol analysis**					
Age at start of trial (years):					
6-9	24.9 (1.0); n=59	24.9 (0.9); n=27		−0.11 (−0.28 to 0.06)	0.65
10-12	24.9 (1.1); n=47	24.9 (0.9); n=30		−0.15 (−0.33 to 0.02)	

CI=confidence interval; D=dioptre; SD=standard deviation.

* Adjusted for baseline spherical equivalent refractive error.

† Mean difference estimate from generalised estimating equation and interaction term P value from a global test for interaction.

‡ Includes light brown, green, blue, and grey.

Table 4 shows the measures of adherence to the study drugs and reasons for withdrawal from the study. Overall adherence to the study eye drops was more than 80% in both groups. More than two thirds of participants (69% in the atropine group and 70% in the placebo group) had good adherence (≥80%) throughout the two year study period.

## Discussion

The results of the CHAMP-UK trial indicate that atropine 0.01% eye drops were well tolerated and reduced the rate of myopia progression in children in a UK population, although the effect was small. The results also indicated that the drops were well tolerated. Changes in refractive error and central axial length, both key quantifiers of myopia, were reduced with the atropine compared with placebo eye drops by a mean of 0.38 D and 0.14 mm, respectively. Additionally, more participants had stable myopia (change less than 0.25 D) in the atropine group than in the placebo group. Fewer participants in the atropine group had clinically significant progression (change more than 0.50 D) than participants in the placebo group, and fewer in the atropine group had fast progression of 1 D or more compared with the placebo group: 16 (16%) versus 24 (42%). The per protocol analysis, facilitated by the electronic measure of adherence (set at ≥80% adherence), showed slightly greater efficacy in the primary outcome and in constraining central axial length. Subgroup analyses exploring the influence of age, ethnicity, severity of myopia, iris colour, and sex did not reveal any statistically significant difference, although our study was not powered to detect differences within subgroups of participants and thus these findings should be interpreted cautiously.

Atropine was well tolerated, with no differences in adverse events between atropine eye drops and placebo. Biological activity was verified through observed changes in pupil size and accommodative ability among participants who received treatment, although these ocular effects were minimal and did not result in reports of light sensitivity or blurred vision at near reading distance.

### Comparison with other studies

Several interventions to reduce the progression of myopia have been investigated, including multifocal contact lenses, orthokeratology, peripheral defocus spectacle lenses, and repeated low dose red light therapy.^42^ A recent Cochrane review synthesised current evidence and suggested that high concentration atropine (≥0.5%) is probably the most effective treatment for myopia, but it is associated with potential side effects such as blurring, impaired reading vision, photophobia, and allergic reactions.^16^ The review included 20 trials of low concentration atropine (<0.1%), most conducted in East Asia, and confirmed that the treatment was well tolerated but efficacy was still uncertain. Studies in Singapore, Hong Kong, and China show an effect of low concentration atropine on axial elongation and myopia control, albeit with some mismatch between reported rates of myopia progression and axial elongation with low concentrations.^20^ ^21^ ^43^ The CHAMP-UK study found no such mismatch between rate of myopia progression and rate of axial elongation. Any myopia control intervention should show reduction in both rate of myopia progression and rate of axial elongation. In previous studies where such a correlation was not found, it could be speculated that penetration of the drug to the retina did not occur or that errors in measurement accounted for this discrepancy.

Low concentration atropine is now widely used in many countries for treating children with myopia and pre-myopia; however, it has not been widely tested in European populations until recently.^22^ ^23^ ^24^ ^25^ As Asian children seem to be more susceptible to the development of myopia and have a faster rate of progression than white European children,^44^ potentially due to cultural differences, the impact of atropine may vary with population studied. In addition, differences may be due to genetic factors, environmental factors such as baseline outdoor time, and nuances in study design contributing to these variances in outcomes.

Although myopia related blindness is uncommon, the risk of visual loss increases with severity of myopia,^45^ and thus there is a need for myopia control interventions to slow progression of the condition in children. Two recent studies have evaluated the long term economic impact of myopia and suggested that interventions to reduce myopia progression are cost effective.^46^ ^47^ The findings from the current study suggest that atropine 0.01% may help reduce long term severe complications associated with myopia.

An international, industry funded study (CHAMP) in mainly European derived populations showed statistically significant but modest efficacy (0.24 D slowing of myopia progression and 0.13 mm difference in axial elongation over three years) with 0.01% atropine eye drops but no significant differences in either refraction or axial length with 0.02% atropine.^25^ It should be noted that CHAMP-UK and CHAMP trials are independent from each other, with different funder, sponsor, and participants. Other recent trials have not found statistically significant differences (table 6).^22^ ^23^ ^24^ A single centre two year trial from Ireland, MOSAIC (Myopia Outcome Study of Atropine in Children), did not find any statistically significant differences in myopia progression between low concentration atropine (0.01%) eye drops and placebo (mean difference 0.10 D, P=0.07) but reported a small, significant reduction in axial length in the atropine group compared with placebo (−0.07 mm, P=0.007).^22^ The authors suggested that results may have been affected by lockdown restrictions during the covid-19 pandemic altering participant behaviour and resulting in faster myopia progression rates. A smaller trial (153 participants, 49% from Europe and 18% of east Asian ancestry) in Western Australia^24^ although finding significant differences in spherical equivalent and axial length at 6, 12, and 18 months, reported a non-significant difference in myopia progression with atropine 0.01% at 24 months (adjusted mean difference 0.14 D, 95% CI −0.03 to 0.29 over two years). The likely explanation for the 24 month lack of significance was that a larger proportion of the placebo group of the study dropped out compared with the treatment group. Both MOSAIC and WA-ATOM recruited children over a wider age range than CHAMP-UK, from 6 to 16 years, perhaps partially explaining differences in findings with our study, given the natural slowing of myopia in older ages. A US based randomised trial (PEDIG study) of atropine 0.01% (the same preservative-free formulation as used in the CHAMP and MOSAIC studies) enrolled 187 children with a more similar participant age profile to CHAMP-UK and found no statistically significant difference between atropine and placebo in terms of myopia progression (mean difference −0.02 D, 95% CI −0.19 to 0.15, P=0.83) over a two year period.^23^ Limitations of previously published studies include smaller sample sizes and a lack of an objective measure of eye drop use, with typically calendar review and parental report used to record adherence to eye drop use. Research indicates potential over-estimation of adherence by these methods compared with an objective assessment of adherence.^48^ ^49^ The effect size in refractive error observed in our study is similar to other trials conducted primarily on Chinese populations evaluating low concentrations of atropine. According to the recent Cochrane review, the mean difference in myopia progression between groups treated with low concentration atropine versus placebo eye drops was 0.25 D (95% CI 0.16 to 0.35), but with very low certainty evidence.^16^ The effect size with respect to axial length reported by the Cochrane review was −0.10 mm (95% CI −0.13 to −0.07), with a very low certainty evidence, a similar value to the effect size reported in the present study.

**Table 6:** Comparison of low concentration atropine (0.01%) studies in European populations. Differences in myopia progression and axial elongation along with study characteristics in intention-to-treat population

Study	Recruitment dates	Age range (years)	Myopia range (D)	Study drug	Study length (years)	Difference (95% CI) in myopia progression (D)	Difference (95% CI) in axial elongation (mm)
CHAMP-UK	Jun 2019 to Feb 2022	6-12	SER ≤−0.50 in both eyes	Preserved 0.01% benzalkonium chloride	2	0.33 (0.17 to 0.49)	−0.14 (−0.21 to −0.07)
MOSAIC^22^	Jul 2019 to Sep 2020	6-16	SER ≤−0.50 in both eyes	Preservative-free single use	2	0.12 (SD (0.07))	−0.07 (SD (0.03))
WA-ATOM^24^	Jun 2017 to Dec 2019	6-16	SER ≤−1.50 with progression ≥0.5 D/year	Preserved 0.01% benzalkonium chloride	2	0.14 (−0.03 to 0.29)	−0.05 (−0.11 to 0.01)
PEDIG^23^	Jun 2018 to Sep 2022	5-12	−1.00 to −6.00 SER	Preservative-free single use	2	−0.02 (−0.19 to 0.15)	−0.002 (−0.11 to 0.102)
CHAMP^25^ USA/Europe	Nov 2017 to Aug 2022	6-10*	−0.50 to −6.00 SER	Preservative-free single use	3	0.24* (0.11 to 0.37)	−0.13* (−0.19 to −0.07)

CI=confidence interval; D=dioptre; SD=standard deviation; SER=spherical equivalent refractive error.

* Modified intention to treat.

### Strengths and limitations of this study

CHAMP-UK was the largest study evaluating 0.01% atropine eye drops, the only one using an electronic adherence monitoring system, and with ATOM-WA the only ones using preserved drugs. The use of MEMS may have influenced the behaviour of the parents and increased adherence in our study; however, it is also possible the formulation and study size may have been contributing factors in the study outcomes. Adherence was suboptimal in nearly one third of participants, but the results of the intention-to-treat analyses were similar to our per protocol results. These findings suggest that the findings are generalisable in terms of efficacy to a UK population.

A limitation of the current study is that as it is an efficacy trial, with adherence assessed by MEMS device, the generalisability of these findings to non-trial settings without the advantage of objective monitoring of adherence is unknown. In addition, subgroup analyses were underpowered to be able to detect differences; tolerability was not assessed with a validated questionnaire; the covid-19 pandemic occurred during the current study timeline and it is not known how this may have impacted the results as disentangling the differences in lifestyle patterns between all study participants was not feasible; and we did not prespecify sex in the statistical analysis plan.

Some open questions remain, such as the long term efficacy of atropine in UK and European children. Participants in our trial will have a five year post-randomisation assessment of the primary outcome (refractive error). Optical interventions to slow myopia progression such as dual focus soft contact lenses, peripheral-plus spectacle lenses, and orthokeratology contact lenses have been available on the UK and Europe markets for more than five years, and the Cochrane meta-analysis suggests that they may have a stronger effect on myopia progression than 0.01% atropine but the efficacy estimates of optical interventions are uncertain, and high quality data from comparative randomised controlled trials of these interventions in UK and European populations are limited. The effectiveness of combined optical and drug treatments, which may provide an augmented effect, deserves further research effort. Alternative technologies, such as light therapy, are also emerging.^50^ ^51^

### Policy implications

Atropine eye drops are widely used worldwide to slow progression of myopia in children, but an atropine 0.01% preparation is not currently available in the UK National Health Service for treating children with myopia. The results of the CHAMP-UK trial will help inform policymakers in the UK.

### Conclusions

Low concentration 0.01% atropine treatment was well tolerated and effective and should be considered as an alternative to manage myopia progression.

**What is already known on this topic**

The prevalence of myopia in children is increasing worldwide, and it is associated with increased healthcare costs and long term ocular complications
Optical and drug interventions to reduce myopia progression have been evaluated, particularly in East Asia, but the efficacy of dilute atropine interventions in European derived populations is uncertain
Atropine eye drops are used worldwide, by ophthalmologists and optometrists, to slow progression of myopia in children, but an atropine 0.01% preparation is not currently available in the UK National Health Service for treating myopia in children

**What this study adds**

In a multicentre placebo controlled, double-masked, randomised trial in the UK, low concentration atropine (0.01%) eye drops were well tolerated and effectively reduced myopia progression in children with myopia compared with placebo eye drops
The findings suggest that a low concentration atropine eye drop product would be a worthwhile addition to currently available optical interventions for the treatment of myopia in children in the UK

## Data Availability

The code used to analyse the data in the paper can be found in the supplemental files. The data underlying the findings in this paper are openly and publicly available and can be found here: https://doi.org/10.25934/PR00013003. If you encounter problems accessing the data, please contact the corresponding author.

## Web extra

Extra material supplied by authors

## Notes

Contributors: AAB and NSL contributed equally. AAB, NSL, CMcD, LM, MMcF, JP, NC, REH, CJH, KS, PMA, NS, IF, JL, DM, SL, and MC contributed to the study design. EMcC, SK, GK, SJ, GO’H, SP, BM, SN, and ADN contributed to data collection. CMcD did the statistical analysis. AAB, NSL, CMcD, NC, REH, CJH, KS, PMA, NS, and MC contributed to data interpretation and drafted the manuscript. All authors contributed to revision of the manuscript and approved the final draft. AAB is the guarantor. The corresponding author attests that all listed authors meet authorship criteria and that no others meeting the criteria have been omitted.

Transparency: The lead authors (the manuscript’s guarantors) affirm that the manuscript is an honest, accurate, and transparent account of the study being reported; that no important aspects of the study have been omitted; and that any discrepancies from the study as planned (and, if relevant, registered) have been explained.

Dissemination to participants and related patient and public communities: A lay summary of the results of the study have been sent to research participants. Dissemination to clinicians will be via peer reviewed publications and ophthalmology, optometry, and medical conference presentations.

Provenance and peer review: Not commissioned; externally peer reviewed.
