# Supplementary material for: Low concentration atropine eye drops and progression of myopia in children: multicentre placebo controlled, double masked, randomised trial in the UK (CHAMP-UK)
Source: BMJ. 2026 Jun 11;393:e086698. doi: 10.1136/bmj-2025-086698 (PMC13274572; doi:10.1136/bmj-2025-086698)
Supplement: Supplementary file 1 — Supplementary information: Additional tables 1-3 [file azua086698.wt1.pdf]

Table 1 Primary and secondary outcomes at other time points

Variables		Treatment Group		Adjusted [a]		Adjusted [b]	
		0.01% Atropine sulfate	Placebo	Mean difference (95% CI)	P value	Mean difference (95% CI)	P value
**Spherical Equivalent Refractive Error**							
**SER ITT [c]**	6 months	-0.03 (0.32) n=164	-0.28 (0.30) n=88	0.22 (0.11 to 0.33)	<0.001	0.23 (0.12 to 0.34)	<0.001
	12 months	-0.14 (0.42) n=152	-0.42 (0.39) n=81	0.26 (0.14 to 0.37)	<0.001	0.27 (0.15 to 0.38)	<0.001
	18 months	-0.27 (0.51) n=151	-0.55 (0.57) n=78	0.25 (0.13 to 0.36)	<0.001	0.25 (0.14 to 0.37)	<0.001
**SER PP [c]**	Baseline	-2.87 (1.65) n=110	-2.77 (1.57) n=62				
	6 months	-0.004 (0.29) n=108	-0.30 (0.30) n=62	0.26 (0.13 to 0.40)	<0.001	0.28 (0.14 to 0.41)	<0.001
	12 months	-0.12 (0.41) n=104	-0.45 (0.36) n=58	0.31 (0.17 to 0.44)	<0.001	0.32 (0.18 to 0.45)	<0.001
	18 months	-0.28 (0.49) n=107	-0.58 (0.52) n=57	0.28 (0.15 to 0.42)	<0.001	0.29 (0.16 to 0.43)	<0.001
**Secondary Outcomes**							
**Reading speed ITT [d]**	6 months	113.0 (29.9) n=168	114.1 (27.7) n=88	-1.8 (-6.4 to 2.8)	0.44		
	12 months	119.0 (29.4) n=158	116.8 (29.7) n=82	1.0 (-3.7 to 5.8)	0.67		
	18 months	125.4 (30.7) n=154	125.0 (29.4) n=79	-1.5 (-6.2 to 3.3)	0.55		
**Reading speed PP [d]**	Baseline	106.1 (29.9) n=113	107.1 (26.3) n=62				
	6 months	111.5 (28.2) n=112	115.5 (27.2) n=62	-3.0 (-8.4 to 2.4)	0.27		
	12 months	118.7 (29.4) n=109	118.6 (26.6) n=59	0.7 (-4.7 to 6.2)	0.79		
	18 months	125.3 (30.0) n=110	126.1 (26.7) n=57	-1.1 (-6.6 to 4.4)	0.69		
**Uniocular BCdVA ITT [c]**	6 months	54.4 (3.9) n=168	54.7 (4.0) n=88	-0.21 (-0.92 to 0.49)	0.55		
	12 months	54.8 (3.7) n=158	54.2 (3.3) n=82	0.50 (-0.22 to 1.21)	0.17		
	18 months	55.1 (3.7) n=155	55.3 (3.4) n=79	-0.05 (-0.77 to 0.67)	0.89		
**Uniocular BCdVA PP [c]**	Baseline	54.0 (3.5) n=113	54.3 (3.6) n=62				
	6 months	54.4 (3.6) n=112	55.2 (3.9) n=62	-0.71 (-1.52 to 0.09)	0.08		
	12 months	55.1 (3.8) n=109	54.6 (3.3) n=59	0.63 (-0.18 to 1.45)	0.13		

	18 months	55.3 (3.6) n=111	55.5 (3.5) n=57	0.07 (-0.75 to 0.89)	0.86		
**Binocular BCdVA ITT** [d]	6 months	56.5 (3.9) n=168	56.8 (3.9) n=88	-0.1 (-0.8 to 0.7)	0.84		
	12 months	57.2 (3.8) n=158	56.9 (3.1) n=82	0.4 (-0.3 to 1.2)	0.27		
	18 months	57.1 (3.6) n=155	57.4 (3.3) n=79	0.03 (-0.7 to 0.8)	0.94		
**Binocular BCdVA PP** [d]	Baseline	56.2 (3.8) n=113	56.9 (3.5) n=62				
	6 months	56.6 (3.6) n=112	57.3 (3.8) n=62	-0.3 (-1.1 to 0.6)	0.51		
	12 months	57.2 (3.9) n=109	57.1 (3.2) n=59	0.5 (-0.3 to 1.4)	0.24		
	18 months	57.2 (3.6) n=111	57.6 (3.4) n=57	0.2 (-0.7 to 1.0)	0.72		
**Central axial length ITT** [c]	6 months	24.6 (1.0) n=168	24.6 (0.9) n=87	-0.08 (-0.15 to -0.01)	0.03		
	12 months	24.7 (1.0) n=158	24.8 (1.2) n=82	-0.15 (-0.22 to -0.07)	<0.001		
	18 months	24.8 (1.0) n=155	24.8 (0.9) n=79	-0.10 (-0.18 to -0.03)	0.007		
**Central axial length PP** [c]	Baseline	24.6 (1.0) n=113	24.4 (0.9) n=62				
	6 months	24.7 (1.0) n=112	24.6 (0.9) n=62	-0.10 (-0.17 to -0.03)	0.003		
	12 months	24.8 (1.0) n=109	24.7 (0.9) n=59	-0.09 (-0.16 to -0.02)	0.01		
	18 months	24.9 (1.0) n=111	24.8 (0.9) n=57	-0.10 (-0.17 to -0.03)	0.004		
**Uniocular near VA ITT**[c]	6 months	68.4 (4.44) n=168	68.9 (3.93) n=88	-0.53 (-1.35 to 0.29)	0.20		
	12 months	69.0 (3.68) n=158	69.3 (3.96) n=82	-0.48 (-1.31 to 0.36)	0.26		
	18 months	69.7 (3.68) n=155	69.8 (3.82) n=79	-0.10 (-0.94 to 0.74)	0.82		
**Uniocular near VA PP**[c]	Baseline	67.7 (4.04) n=113	68.0 (4.28) n=61				
	6 months	68.5 (4.26) n=112	69.1 (4.00) n=62	-0.45 (-1.41 to 0.51)	0.36		
	12 months	69.0 (3.93) n=109	69.5 (3.84) n=59	-0.39 (-1.36 to 0.58)	0.43		
	18 months	69.8 (3.56) n=111	70.2 (3.71) n=57	-0.17 (-1.14 to 0.81)	0.74		
**Binocular near VA ITT** [d]	6 months	70.5 (4.40) n=168	71.2 (4.26) n=88	-0.63 (-1.47 to 0.20)	0.14		
	12 months	71.3 (3.62) n=158	71.5 (3.79) n=82	-0.27 (-1.13 to 0.59)	0.54		
	18 months	71.8 (3.27) n=155	72.1 (3.51) n=79	-0.13 (-1.00 to 0.74)	0.77		
	Baseline	70.2 (3.68) n=113	70.8 (4.34) n=61				

**Binocular near VA PP** [d]	6 months	70.5 (4.14) n=112	71.3 (4.41) n=62	-0.62 (-1.59 to 0.34)	0.21		
	12 months	71.3 (3.65) n=109	71.8 (3.88) n=59	-0.25 (-1.23 to 0.73)	0.62		
	18 months	72.0 (2.93) n=111	72.4 (3.39) n=57	0.01 (-0.98 to 1.00)	0.98		
**Pupil diameter ITT** [c]	6 months	6.71 (0.90) n=167	6.11 (0.90) n=86	0.58 (0.42 to 0.74)	<0.001		
**Pupil diameter PP** [c]	Baseline	6.07 (0.87) n=113	6.11 (0.84) n=62				
	6 months	6.82 (0.85) n=111	6.08 (0.89) n=61	0.77 (0.60 to 0.95)	<0.001		
	12 months	6.82 (0.71) n=108	6.11 (0.81) n=59	0.74 (0.56 to 0.92)	<0.001		
	18 months	6.74 (0.78) n=111	6.23 (0.91) n=57	0.53 (0.35 to 0.70)	<0.001		
**Spectacle correction ITT** [e]	6 months	167 (99.4%)	88 (100.0%)				
	12 months	158 (100.0%)	82 (100.0%)				
	18 months	154 (99.4%)	78 (98.7%)				
**Spectacle correction PP** [e]	Baseline	113 (100.0%)	60 (96.8%)				
	6 months	112 (100.0%)	62 (100.0%)				
	12 months	109 (100.0%)	59 (100.0%)				
	18 months	110 (99.1%)	57 (100.0%)				
**Frequency of spectacle correction (all day) ITT** [e]	6 months	135 (81.3%)	68 (77.3%)				
	12 months	130 (82.3%)	65 (79.3%)				
	18 months	131 (85.1%)	65 (83.3%)				
**Frequency of spectacle correction (all day) PP** [e]	Baseline	88 (77.9%)	46 (76.7%)				
	6 months	94 (83.9%)	46 (74.2%)				
	12 months	93 (85.3%)	47 (79.7%)				
	18 months	97 (88.2%)	48 (84.2%)				
**Frequency of spectacle correction (sometimes) ITT** [e]	6 months	31 (18.7%)	20 (22.7%)				
	12 months	28 (17.7%)	17 (20.7%)				
	18 months	23 (14.9%)	13 (16.7%)				
**Frequency of spectacle correction (sometimes) PP** [e]	Baseline	25 (22.1%)	14 (23.3%)				
	6 months	18 (16.1%)	16 (25.8%)				
	12 months	16 (14.7%)	12 (20.3%)				
	18 months	13 (11.8%)	9 (15.8%)				
**EQ-5D-Y VAS**	6 months	94.1 (9.6) n=168	94.8 (7.6) n=88	-0.6 (-2.4 to 1.3)	0.56		

**ITT** [d]	12 months	95.0 (6.5) n=157	94.8 (7.2) n=82	0.1 (-1.9 to 2.0)	0.93		
	18 months	94.7 (7.5) n=155	94.2 (6.9) n=79	0.4 (-1.5 to 2.4)	0.66		
**EQ-5D-Y VAS PP** [d]	Baseline	95.9 (6.5) n=113	96.3 (6.4) n=62				
	6 months	94.5 (8.1) n=112	94.7 (8.4) n=62	-0.1 (-2.1 to 2.0)	0.96		
	12 months	95.7 (5.1) n=108	95.7 (6.0) n=59	0.02 (-2.0 to 2.1)	0.98		
	18 months	95.2 (6.7) n=111	94.8 (6.1) n=57	0.6 (-1.5 to 2.6)	0.59		
**Tolerability**							
**Eye feels ITT** [f]	6 months	3.6 (0.5) n=166	3.6 (0.5) n=87	0.01 (-0.1 to 0.1)	0.91		
	12 months	3.7 (0.5) n=157	3.7 (0.5) n=81	-0.06 (-0.2 to 0.1)	0.39		
	18 months	3.7 (0.5) n=153	3.8 (0.4) n=79	-0.08 (-0.2 to 0.1)	0.28		
**Eye feels PP** [f]	6 months	3.6 (0.5) n=112	3.7 (0.5) n=62	-0.05 (-0.2 to 0.1)	0.50		
	12 months	3.6 (0.5) n=109	3.7 (0.5) n=59	-0.10 (-0.3 to 0.1)	0.20		
	18 months	3.7 (0.5) n=111	3.9 (0.4) n=57	-0.15 (-0.3 to 0.01)	0.06		
**Stingy ITT** [f]	6 months	3.1 (0.9) n=167	3.0 (1.0) n=87	0.1 (-0.1 to 0.4)	0.31		
	12 months	3.3 (0.9) n=157	3.3 (0.8) n=81	-0.09 (-0.3 to 0.2)	0.48		
	18 months	3.4 (0.9) n=150	3.3 (0.9) n=75	0.06 (-0.2 to 0.3)	0.66		
**Stingy PP** [f]	6 months	3.2 (0.9) n=112	3.1 (1.0) n=62	0.1 (-0.2 to 0.4)	0.41		
	12 months	3.2 (0.9) n=109	3.4 (0.7) n=59	-0.2 (-0.4 to 0.1)	0.25		
	18 months	3.4 (0.8) n=110	3.4 (0.8) n=56	0.04 (-0.2 to 0.3)	0.79		
**Itchiness ITT** [f]	6 months	3.4 (0.9) n=166	3.3 (1.0) n=86	0.1 (-0.1 to 0.3)	0.37		
	12 months	3.4 (0.9) n=157	3.5 (0.7) n=81	-0.08 (-0.3 to 0.1)	0.50		
	18 months	3.5 (0.7) n=152	3.4 (0.8) n=79	0.1 (-0.1 to 0.3)	0.38		
**Itchiness PP** [f]	6 months	3.5 (0.8) n=112	3.4 (0.9) n=61	0.1 (-0.1 to 0.4)	0.33		
	12 months	3.4 (0.9) n=109	3.6 (0.6) n=59	-0.3 (-0.5 to -0.001)	0.049		
	18 months	3.5 (0.7) n=111	3.5 (0.7) n=57	0.01 (-0.2 to 0.3)	0.94		
**Blurry Vision ITT** [f]	6 months	3.6 (0.9) n=166	3.5 (1.0) n=86	0.2 (-0.03 to 0.3)	0.10		
	12 months	3.7 (0.6) n=156	3.8 (0.5) n=80	-0.01 (-0.2 to 0.2)	0.95		
	18 months	3.8 (0.6) n=152	3.8 (0.6) n=79	-0.003 (-0.2 to 0.2)	0.97		
**Blurry Vision PP** [f]	6 months	3.6 (0.9) n=112	3.5 (1.0) n=61	0.1 (-0.1 to 0.4)	0.25		
	12 months	3.7 (0.7) n=109	3.8 (0.4) n=59	-0.08 (-0.3 to 0.2)	0.50		
	18 months	3.8 (0.6) n=111	3.8 (0.5) n=57	-0.04 (-0.3 to 0.2)	0.76		
**Eyes sore ITT** [f]	6 months	3.3 (1.0) n=164	3.3 (0.9) n=86	0.02 (-0.2 to 0.3)	0.88		
	12 months	3.3 (1.0) n=156	3.5 (0.7) n=80	-0.1 (-0.4 to 0.1)	0.22		
	18 months	3.3 (0.9) n=153	3.5 (0.7) n=79	-0.2 (-0.4 to 0.1)	0.18		

**Eyes sore PP** [f]	6 months	3.3 (0.9) n=111	3.4 (0.9) n=61	-0.04 (-0.3 to 0.2)	0.76		
	12 months	3.2 (1.0) n=109	3.5 (0.7) n=59	-0.3 (-0.6 to -0.03)	0.03		
	18 months	3.3 (0.9) n=111	3.6 (0.8) n=57	-0.3 (-0.6 to -0.02)	0.04		
**Difficult to read/write ITT** [f]	6 months	3.6 (0.9) n=165	3.5 (1.0) n=86	0.1 (-0.1 to 0.3)	0.29		
	12 months	3.7 (0.8) n=156	3.8 (0.6) n=81	-0.1 (-0.3 to 0.1)	0.25		
	18 months	3.8 (0.7) n=152	3.8 (0.6) n=79	-0.04 (-0.3 to 0.2)	0.68		
**Difficult to read/write PP** [f]	6 months	3.7 (0.9) n=111	3.5 (1.0) n=62	0.1 (-0.1 to 0.4)	0.31		
	12 months	3.6 (0.8) n=109	3.8 (0.6) n=59	-0.2 (-0.5 to 0.02)	0.07		
	18 months	3.8 (0.7) n=111	3.9 (0.4) n=57	-0.1 (-0.4 to 0.1)	0.30		

[a] Adjusted for baseline. [b] Adjusted for baseline SER, minimisation variables (i.e. site and ethnicity), age and history of parental myopia (at least one parent). [c] Mean(SD) was calculated with data from both eyes. Mean difference (CIs) and p value from repeated measures mixed model. [d] Mean difference (CIs) and p value from repeated measures mixed model. [e] Frequency (%) presented for categorical variables [f] Mean difference (CIs) and p value from repeated measures mixed model, no adjustment for baseline as tolerability not measured at baseline.

Table 2 Safety outcomes by treatment group

	Number of Events			Number of Patients				
	**Total**	**Atropine sulfate**	**Placebo**	**Total N=289**	**Atropine sulfate N=192**	**Placebo N=97**	**Risk Ratio (95% CI)**	**p-value**
**Adverse Events**	162	114 (70.4%)	48 (29.6%)	98	70 (36.5%)	28 (28.9%)	1.3 (0.9 to 1.8)	0.24
**Adverse Reactions**	42	38 (90.5%)	4 (9.5%)	32	28 (14.6%)	4 (4.1%)	3.5 (1.3 to 9.8)	<0.01*
**Unexpected Adverse Reactions**	2	2 (100%)	0 (0.0%)	2	2 (1.0%)	0 (0.0%)	NA	NA
**Serious Adverse Events**	7	6 (85.7%)	1 (14.3%)	6	5 (2.6%)	1 (1.0%)	2.5 (0.3 to 21.3)	0.67
**Serious Adverse Reactions**	0	0 (0.0%)	0 (0.0%)	0	0 (0.0%)	0 (0.0%)	NA	NA

**Suspected Unexpected Serious Adverse Reactions**	0	0 (0.0%)	0 (0.0%)	0	0 (0.0%)	0 (0.0%)	NA	NA
**Fatal Serious Adverse Events**	0	0 (0.0%)	0 (0.0%)	0	0 (0.0%)	0 (0.0%)	NA	NA

*specific p-value for Adverse Reactions: P = 0.009

Table 3 Safety outcomes by system organ class and treatment group

		Number of Events			Number of Patients				
		**Total**	**Atropine sulfate**	**Placebo**	**Total N=289**	**Atropine sulfate N=192**	**Placebo N=97**	**RR (95%CI)**	**p-value**
**AEs**	Ear and labyrinth disorders	1	1 (100.0%)	0 (0.0%)	1	1 (0.5%)	0 (0.0%)	NA	NA
	Endocrine disorders	1	0 (0.0%)	1 (100.0%)	1	0 (0.0%)	1 (1.0%)	NA	NA

		Number of Events			Number of Patients				
		Total	Atropine sulfate	Placebo	Total N=289	Atropine sulfate N=192	Placebo N=97	RR (95%CI)	p-value
	Eye disorders	79	63 (79.8%)	16 (20.3%)	54	42 (21.9%)	12 (12.4%)	1.8 (1.0 to 3.2)	0.06
	Gastrointestinal disorders	8	5 (62.5%)	3 (37.5%)	8	5 (2.6%)	3 (3.1%)	0.8 (0.2 to 3.5)	1.00
	General disorders and administration site conditions	2	0 (0.0%)	2 (100.0%)	2	0 (0.0%)	2 (2.1%)	NA	NA
	Infections and infestations	30	18 (60.0%)	12 (40.0%)	26	16 (8.3%)	10 (10.3%)	0.8 (0.4 to 1.7)	0.66
	Injury, poisoning and procedural complications	2	2 (100.0%)	0 (0.0%)	2	2 (1.0%)	0 (0.0%)	NA	NA
	Musculoskeletal and connective tissue disorders	5	3 (60.0%)	2 (40.0%)	4	3 (1.6%)	1 (1.0%)	1.5 (0.2 to 14.4)	1.00
	Nervous system disorders	18	10 (55.6%)	8 (44.4%)	14	8 (4.2%)	6 (6.2%)	0.7 (0.2 to 1.9)	0.56
	Reproductive system and breast disorders	1	1 (100.0%)	0 (0.0%)	1	1 (0.5%)	0 (0.0%)	NA	NA
	Respiratory, thoracic and mediastinal disorders	8	6 (75.0%)	2 (25.0%)	8	6 (3.1%)	2 (2.1%)	1.5 (0.3 to 7.4)	0.72

		Number of Events			Number of Patients				
		Total	Atropine sulfate	Placebo	Total N=289	Atropine sulfate N=192	Placebo N=97	RR (95%CI)	p-value
	Skin and subcutaneous tissue disorders	7	5 (71.4%)	2 (28.6%)	7	5 (2.6%)	2 (2.1%)	1.3 (0.2 to 6.4)	1.00
**ARs**	Eye disorders	41	37 (90.2%)	4 (9.8%)	31	27 (14.1%)	4 (4.1%)	3.4 (1.2 to 9.5)	<0.01*
	Skin and subcutaneous tissue disorders	1	1 (100.0%)	0 (0.0%)	1	1 (0.5%)	0 (0.0%)	NA	NA
**UARs**	Eye disorders	1	1 (100.0%)	0 (0.0%)	1	1 (0.5%)	0 (0.0%)	NA	NA
	Skin and subcutaneous tissue disorders	1	1 (100.0%)	0 (0.0%)	1	1 (0.5%)	0 (0.0%)	NA	NA
**SAEs**	Eye disorders	2	2(100.0%)	0 (0.0%)	1	1 (0.5%)	0 (0.0%)	NA	NA
	Gastrointestinal disorders	2	1 (50.0%)	1 (50.0%)	2	1 (0.5%)	1 (1.0%)	0.5 (0.0 to 8.0)	1.00
	Infections and infestations	1	1 (100.0%)	0 (0.0%)	1	1 (0.5%)	0 (0.0%)	NA	NA
	Reproductive system and breast disorders	1	1 (100.0%)	0 (0.0%)	1	1 (0.5%)	0 (0.0%)	NA	NA
	Skin and subcutaneous tissue disorders	1	1 (100.0%)	0 (0.0%)	1	1 (0.5%)	0 (0.0%)	NA	NA

		Number of Events			Number of Patients				
		**Total**	**Atropine sulfate**	**Placebo**	**Total N=289**	**Atropine sulfate N=192**	**Placebo N=97**	**RR (95%CI)**	**p-value**
**SARs**	NA	0	0 (0.0%)	0 (0.0%)	0	0 (0.0%)	0 (0.0%)	NA	NA
**SUSARs**	NA	0	0 (0.0%)	0 (0.0%)	0	0 (0.0%)	0 (0.0%)	NA	NA

*Specific p-value for ARs Eye disorders is 0.009
